# Aging, mitochondrial dysfunction, and cerebral microhemorrhages: a preclinical evaluation of SS-31 (elamipretide) and development of a high-throughput machine learning-driven imaging pipeline for cerebromicrovascular protection therapeutic screening

**DOI:** 10.1007/s11357-025-01634-5

**Published:** 2025-04-02

**Authors:** Roland Patai, Krish Patel, Boglarka Csik, Rafal Gulej, Raghavendra Y. Nagaraja, Dorina Nagy, Siva Sai Chandragiri, Santny Shanmugarama, Kiana Vali Kordestan, Mark Nagykaldi, Shoba Ekambaram, Anna Ungvari, Andriy Yabluchanskiy, Stefano Tarantini, Zoltan Benyo, Anna Csiszar, Zoltan Ungvari, Adam Nyul-Toth

**Affiliations:** 1 Vascular Cognitive Impairment, Neurodegeneration, and Healthy Brain Aging Program, Department of Neurosurgery, University of Oklahoma Health Sciences Center, Oklahoma City, OK USA; 2 Oklahoma Center for Geroscience and Healthy Brain Aging, University of Oklahoma Health Sciences Center, Oklahoma City, OK USA; 3 Institute of Preventive Medicine and Public Health, Semmelweis University, Budapest, Hungary; 4 International Training Program in Geroscience, Doctoral College, Health Sciences Division/Institute of Preventive Medicine and Public Health, Semmelweis University, Budapest, Hungary; 5 The Peggy and Charles Stephenson Cancer Center, University of Oklahoma Health Sciences Center, Oklahoma City, OK USA; 6 International Training Program in Geroscience, Institute of Translational Medicine, Semmelweis University, Budapest, Hungary; 7 Cerebrovascular and Neurocognitive Disorders Research Group, Hungarian Research Network, Semmelweis University (HUN-REN-SU), Budapest, Hungary

**Keywords:** Aging, Cerebral small vessel disease, CSVD, Oxidative stress, Mitochondrial dysfunction, Cerebrovascular protection, VCID, Microbleed, Mitochondria, Electron transport chain

## Abstract

Cerebral microhemorrhages (CMHs, also known as cerebral microbleeds) contribute to vascular cognitive impairment and dementia (VCID), with aging and hypertension being key risk factors. Mitochondrial oxidative stress is a hallmark of cerebrovascular aging, leading to endothelial dysfunction. This study tests the hypothesis that increased mitochondrial oxidative stress contributes to age-related CMH susceptibility and evaluates the mitochondrial-targeted antioxidative peptide SS-31 (elamipretide) as a potential protective agent in an aged, hypertensive mouse model. Concurrently, we developed a high-throughput, machine learning–driven imaging pipeline to enhance CMH quantification and facilitate the screening of anti-aging vasoprotective interventions. To detect CMHs, brain sections were labeled with diaminobenzidine (DAB) and digitized using a slide scanner-based imaging platform. We developed multiple quantification tools, including color space transformation for enhanced contrast separation and a supervised machine-learning approach utilizing a random forest algorithm to generate whole-brain 3D reconstructions and precisely localize CMHs. We optimized a semi-automated detection method integrating color space transformation and machine learning, benchmarking it against traditional manual counting and color deconvolution-based approaches. While SS-31 treatment did not significantly mitigate hypertension-induced CMH burden in aged mice, our high-throughput imaging pipeline provided a reliable, scalable, and unbiased approach to CMH detection, reducing processing time while improving accuracy. This methodological advancement paves the way for future preclinical studies evaluating therapeutic strategies for cerebrovascular protection in aging. Our findings underscore the need for multi-targeted interventions to mitigate CMH-related neurovascular impairments and prevent VCID.

**Supplementary Information:**

The online version contains supplementary material available at 10.1007/s11357-025-01634-5.

## Introduction

Cerebral microhemorrhages (CMHs) are increasingly recognized as contributors to vascular cognitive impairment and dementia (VCID) [1–5]. CMHs are small, focal brain bleeds resulting from the rupture of fragile microvessels, often associated with cerebral small vessel disease (CSVD) [6, 7]. Clinically, CMHs are detected using susceptibility-weighted MRI (SWI) and gradient-echo T2*-weighted imaging, which allow visualization of hemosiderin deposits indicative of chronic microvascular injury [6, 8–14]. CMHs are linked to an increased risk of cognitive decline and stroke. Additionally, CMHs are implicated in Alzheimer’s disease (AD) pathogenesis [11, 15–17].

Aging and hypertension are the two primary risk factors for CMH development [18–23]. Chronic hypertension imposes mechanical stress on the cerebral microvasculature, increasing the risk of rupture, particularly in the context of aging [24–26]. Age-related microvascular degeneration, characterized by endothelial dysfunction, pathological extracellular matrix remodeling, and loss of vascular elasticity, further compromises microvascular integrity, heightening susceptibility to CMHs that eventually leads to neurological symptoms [17, 23, 27–31]. Despite their clinical significance, the precise mechanisms underlying CMH formation remain incompletely understood, and no effective preventive treatments are currently available [32].

To address this gap, we developed a clinically relevant mouse model of CMHs by inducing chronic hypertension in aged mice using angiotensin II (Ang II) and the NO synthase inhibitor N(ω)-nitro-l-arginine methyl ester (L-NAME) [17, 18, 33–36]. Using this model, we previously demonstrated that advanced age substantially exacerbates hypertension-induced CMH burden [18, 24, 30, 32]. Furthermore, our studies revealed that pretreatment with the potent antioxidative polyphenol compound resveratrol reduced CMH formation, suggesting that age-related microvascular fragility involves oxidative stress–driven matrix metalloproteinase (MMP) activation [18, 31]. Mechanistically, we identified increased vascular reactive oxygen species (ROS) production in aged mice, originating from both NADPH oxidase activation and mitochondrial sources [37, 38].

Mitochondrial oxidative stress is a hallmark of vascular aging and plays a central role in endothelial dysfunction [39–41]. Our prior work demonstrated that increased mechanical pressure in aged vessels leads to excessive mitochondrial ROS production, likely contributing to endothelial dysfunction and vascular instability [42]. Additionally, endothelial mitochondrial oxidative stress has been implicated in cerebrovascular dysfunction and BBB disruption in aging [43–47]. Given these findings, targeting mitochondrial oxidative stress represents a promising therapeutic strategy for mitigating CMH burden in aging [48, 49].

SS-31 (elamipretide) is a mitochondria-targeting tetrapeptide (d-Arg-2′,6′-dimethylTyr-Lys-Phe-NH2) that binds to cardiolipin, stabilizing mitochondrial membrane integrity, improving electron transport chain efficiency, and reducing mitochondrial ROS production [50–57]. SS-31 has been shown to reverse mitochondrial dysfunction in various models of cardiovascular and neurodegenerative diseases and has demonstrated protective effects against mitochondrial oxidative stress in the heart and brain [37, 58–62]. Given the lack of clinically relevant CMH prevention treatments, this study serves a dual purpose: (1) evaluating SS-31 as a protective intervention in an aged hypertensive mouse model and (2) developing a scalable high-throughput imaging pipeline integrating machine learning to enhance CMH detection and quantification. By establishing an automated approach, we aim to facilitate the screening of additional geroprotective interventions in relevant preclinical models.

## Materials and methods

### Animals and SS-31 treatment

Young (3-month-old) and aged (24-month-old) male C57BL/6 inbred mice (Jackson Laboratories) were used in this study. The animals were housed under specific pathogen-free barrier conditions in the Rodent Barrier Facility at the University of Oklahoma Health Sciences Center, maintained on a 12-h light/dark cycle with unrestricted access to water and a standard AIN-93G diet.

The young mice served as the control group (*n* = 3), while aged mice (*n* = 6) were divided into two subgroups. Half of the aged cohort remained untreated (*n* = 3), while the other half (*n* = 3) received daily intraperitoneal injections of SS-31 (Elamipretide, also known as MTP-131 or Bendavia; 10 mg/kg/day). SS-31 treatment was initiated 10 days before hypertension induction and maintained throughout the experimental period (see below). This SS-31 dosage has been previously demonstrated to exert significant endothelial protective effects in aged mice [58].

The number of animals per group was determined based on our prior studies assessing CMH burden in aged hypertensive mice, where a similar sample size per group provided sufficient statistical power to detect significant differences using histological quantification [17, 18, 23, 35, 36, 63]. Given the strong effect sizes observed in previous work, we used the same sample size to ensure comparability while adhering to the 3R principles (Replacement, Reduction, and Refinement) to minimize animal use. While larger cohorts may provide additional granularity, our findings align with established methodologies in preclinical vascular aging studies. All experimental procedures were conducted in accordance with protocols approved by the Institutional Animal Care and Use Committee of the University of Oklahoma Health Sciences Center.

### Hypertension induction and cerebral microhemorrhage modeling

Arterial hypertension was induced in aged mice using a combination of angiotensin II (Ang-II) infusion and L-NAME administration [17, 18, 33–36]. Ang-II was delivered subcutaneously via osmotic mini-pumps (Alzet Model 2006, 0.15 µL/h, Durect Co., Cupertino, CA), while L-NAME (100 mg/kg/day; Sigma-Aldrich, St. Louis, MO, USA) was provided in drinking water to further impair endothelial function.

For pump implantation, mice were anesthetized with isoflurane (2.0–2.5%, 1 L/min flow rate; Covetrus, Portland, ME, USA), and a small incision was made in the interscapular region under aseptic conditions. The osmotic mini-pump was inserted into the subcutaneous space, and the incision was closed using surgical sutures. Ang-II was continuously delivered at a rate of 1 µg/min/kg (Sigma-Aldrich).

Blood pressure was monitored using a non-invasive tail-cuff system (CODA Non-Invasive Blood Pressure System, Kent Scientific Co., Torrington, CT, USA), as previously described. To assess the occurrence of clinically manifest microhemorrhages, mice underwent daily neurological examinations following established protocols [17, 18, 23, 25, 32, 35, 36, 64, 65].

### Histological processing and staining

Mice were anesthetized and subjected to transcardial perfusion with ice-cold heparinized PBS for 10 min, followed by decapitation, as previously described [18, 36]. The brains were carefully extracted and initially fixed in 10% formalin (Sigma-Aldrich) at room temperature for 24 h. Subsequently, the samples were transferred to fresh 10% formalin at 4°C for an additional 48 h, followed by immersion in 70% ethanol at 4°C for 2 days to enhance tissue preservation. The brains then underwent a graded ethanol dehydration series before wax infiltration and paraffin embedding for histological analysis.

For sectioning, 8-μm-thick coronal slices were obtained using a HistoCore Autocut R rotary microtome (Leica, Nussloch, Germany). To ensure comprehensive sampling, every two consecutive sections were retained while three were discarded, yielding approximately 1000 sections per brain.

**Fig. 1:**
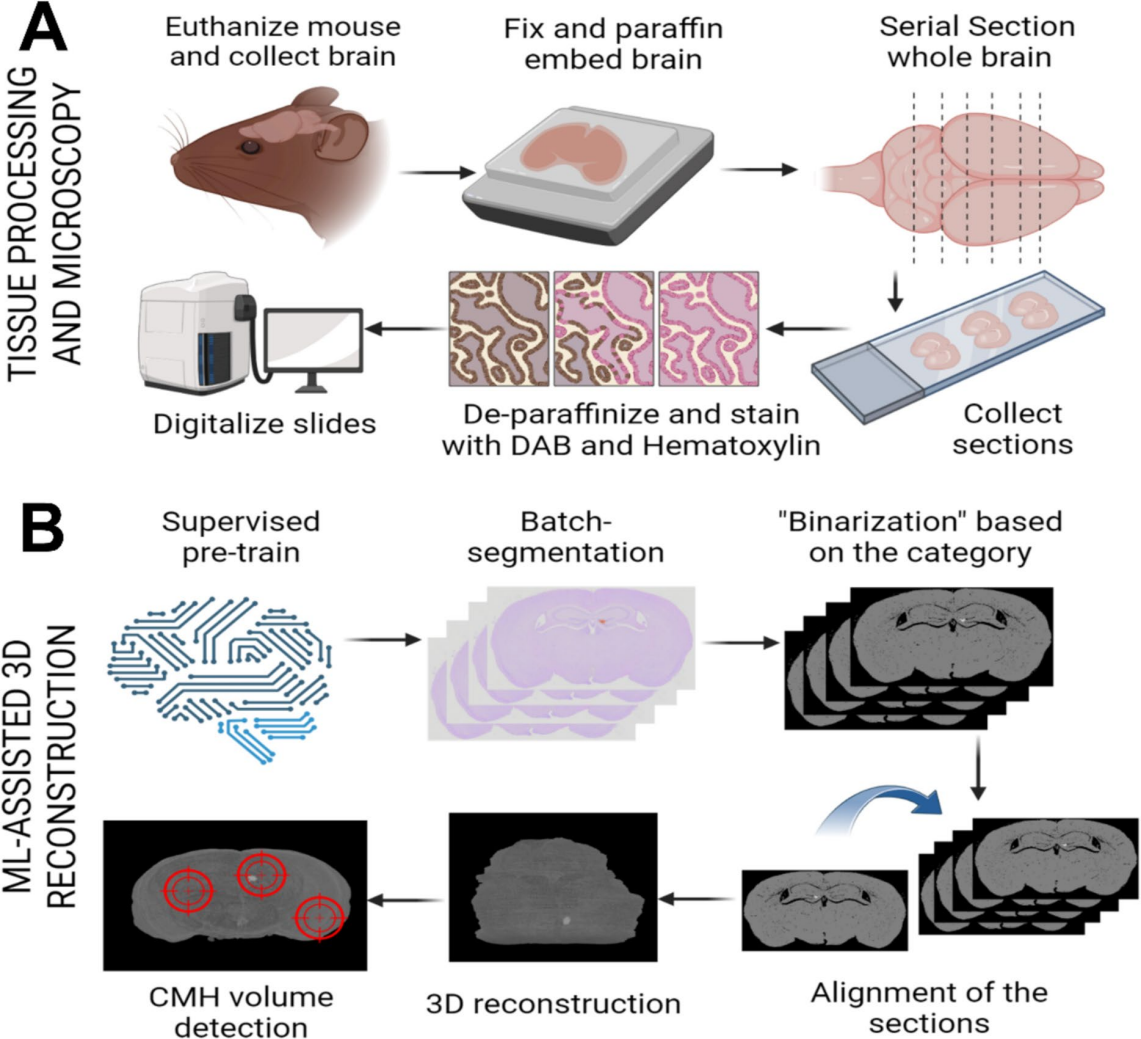
High-throughput machine learning-based pipeline for cerebral microhemorrhage (CMH) detection and quantification. Representative pipeline for high-throughput CMH detection and quantification. **A** Overview of the histological processing, staining, and imaging methods used for CMH analysis. Brain tissues were embedded in paraffin, systematically sectioned, and stained with hematoxylin and diaminobenzidine (DAB) before being digitized using a slide scanner for high-resolution imaging. **B** Digital micrographs of coronal brain sections were manually annotated to create a training dataset for a random forest-based supervised machine learning model. Following model pre-training, images were processed using batch segmentation and anatomical alignment procedures. This was followed by three-dimensional reconstruction to enable the quantification of CMH area and density

Histological staining was performed to facilitate the visualization of both brain structures and microhemorrhages. Sections were stained with Gill No.1 hematoxylin solution (Sigma-Aldrich) to delineate anatomical features and with the ImmPACT diaminobenzidine (DAB) kit (Vector Laboratories, New Ark, CA, USA) to detect hemorrhages (Fig. 1A). DAB staining produces a distinct dark brown precipitate in the presence of peroxidases found in red blood cells, enabling precise identification of extravasated blood cells within the brain parenchyma [18].

All stained sections were examined by a histological expert blinded to the treatment groups to ensure unbiased analysis.

### Slide scanning and manual quantification

Slides were digitized using an Axioscan 7 slide scanner (Zeiss, Jena, Germany) in brightfield mode with a 40 × objective and saved in Zeiss proprietary CZI format. To extract individual sections, digital slides were processed in Zen Blue 3.2 (Zeiss) using the “Split Scene” tool, followed by the “Image Export” function to save the images in TIF format. To optimize computational efficiency, images were downscaled to 20% of their original size while preserving resolution for further analysis.

The downscaled, unprocessed images were used for the manual assessment of CMH incidence in brain tissue. Identified microbleeds were manually segmented and analyzed using ImageJ 1.54f (NIH, Bethesda, MD, USA). The segmentation process involved binarization using the Otsu method, followed by quantification of the number and area of microbleeds using the “Analyze Particles” tool. The extracted CMH data were exported to Excel to calculate the average number of CMHs per section for further statistical analysis.

### Semi-automated image analysis using color deconvolution and color space transformation

Semi-automated image analysis methods using color deconvolution or color space transformation are techniques that were employed to segment and quantify CMHs in stained histological images. These methods leverage computational tools to segment and quantify features of interest in images (CMHs), reducing the manual effort while allowing user input to fine-tune segmentation accuracy.

Color deconvolution is a mathematical technique that separates different color components in histological images, particularly those stained with chromogens like hematoxylin and DAB. This approach isolates individual stains corresponding to specific biomolecules (e.g., RBC-derived peroxidases), facilitating precise microbleed detection.

Color space transformation converts images into alternative color representations (e.g., grayscale, YIQ) to enhance contrast, simplify segmentation, and improve the isolation of stained components. By optimizing contrast, this method enables threshold-based segmentation to distinguish CMHs from background structures.

#### Color deconvolution method

Downscaled and unprocessed Zen Blue 3.2 (Zeiss) TIF files were used for semi-automated analysis. An ImageJ macro automated segmentation of DAB-stained regions by applying color deconvolution using the “HDAB” vector, separating hematoxylin and DAB-stained components. The DAB channel was extracted, followed by a bandpass filter (size parameters: 50 for large structures, 2 for small structures) to suppress high-frequency noise while preserving biologically relevant features.

Next, an automatic threshold using the “Otsu” method was applied to establish segmentation boundaries based on intensity distribution. The resulting image was binarized (“Convert to Mask” function), converting pixel intensities into binary values (0 for background, 255 for segmented CMHs), ensuring clear separation of microbleeds from non-stained regions. The “Analyze Particles” tool was then used to quantify the number and area of CMHs, and the data were exported to Excel for further statistical evaluation.

#### Color space transformation method

Similarly, downscaled TIF files were analyzed using a color space transformation–based pipeline. An ImageJ macro automated segmentation by converting 8-bit indexed color images to the YIQ color space using the “Color Transformer 2” plugin. In this model, the I channel (in-phase chrominance representing orange-blue hues) was selected for further analysis, as it enhances the contrast between hematoxylin and DAB-stained regions.

The Otsu method was then applied for automatic thresholding of the I channel, followed by binarization (“Convert to Mask” function) to ensure a clear distinction between CMHs and surrounding tissue. The “Analyze Particles” tool was used to quantify microbleed count and area, with results exported to Excel for comprehensive analysis.

These semi-automated methods enhance CMH detection accuracy while significantly reducing manual labor, providing a scalable approach for high-throughput histological analysis.

### Automated image processing and machine learning pipeline using LABKIT random forest classifier

Automated image analysis for the detection, localization, and quantification of cerebral microhemorrhages (CMHs) in brain tissue was performed using ImageJ 1.54f (NIH), LABKIT, and custom-made macros, in combination with the StackReg plugin and Imaris 10.0.1 (Oxford Instruments, Abingdon, UK) (Fig. 1B).

#### Training the LABKIT segmentation model

To develop a robust segmentation model, sections from randomized brain regions containing CMHs of varying sizes, structural complexity, and background noise were selected for supervised learning. The LABKIT model was trained to segment three key components: background, brain tissue, and microbleeds.

Background segmentation was optimized by annotating areas near tissue boundaries and intracerebral gaps to prevent misclassification. Microbleed segmentation was refined by annotating both darker regions within the tissue and microbleeds, ensuring differentiation from noise and normal brain structures. To enhance model sensitivity to smaller microbleeds (CMH size < 50 µm), training datasets included approximately 20 small CMHs and 10 larger CMHs (size > 100 µm), with a bias toward smaller microbleeds to prevent exclusion during classification. A diverse dataset was used to prevent overfitting and ensure accurate segmentation across various anatomical regions.

Once the segmentation model was trained, the LABKIT batch segmentation tool was used to process the entire image set. Custom-made ImageJ macros were employed as a comprehensive suite of tools for batch processing and analysis of large histological image datasets of brain tissues with CMHs, including automated file renaming, segmentation, image alignment, and overlaying images onto a standard background.

#### Automated image preprocessing and batch segmentation

A Python-based macro was implemented to scan input directories, identify TIF image files, extract numerical identifiers from filenames, and systematically rename and organize images for downstream analysis. The macro retained the original folder structure, facilitating structured dataset management.

Following file organization, an ImageJ macro was designed to isolate regions of interest (CMHs) via threshold-based binarization. Images were automatically opened, and an adaptive dark threshold was applied to enhance CMH visibility while suppressing background noise. The thresholded image was binarized using the “Convert to Mask” function, reducing pixel values to 0 (background) and 255 (microbleeds/tissue). Further refinement involved converting the images back to 8-bit format and applying the “Fill Holes” operation to smooth segmentation and eliminate artifacts. The processed masks were exported in TIF format, ensuring consistency across datasets for quantitative analysis.

#### Segmentation refinement and image alignment

To improve segmentation accuracy, the LABKIT batch segmentation tool was used to process entire image sets following the initial segmentation phase. Custom ImageJ macros were applied to normalize pixel intensities from the standard 0–255 (8-bit format) to a simplified 0–2 range, representing three segmented regions (0-background, 1-tissue, 2-bleed). Image size standardization was achieved by selecting the largest image, removing segmentation artifacts, and centering each image on a black background to ensure uniform dimensions across the dataset. Due to memory constraints when handling large datasets, a virtual stack was used to crop redundant background regions and reduce data load. The images were then roughly aligned using the “StackReg” plugin, which used the preceding image as a reference for alignment. To maintain consistent registration, another custom macro processed the images in batches of twelve, incorporating two images from the previous batch as references for continued alignment across the dataset. The alignment macros further optimized batch registration by segmenting image sequences into smaller groups, ensuring consistent alignment while minimizing memory usage. The first batch of ten images was aligned independently using rigid body transformation from the “StackReg” tool, and the final two images from the batch are retained as references for subsequent batches. Each new group of images was concatenated with these reference images, ensuring consistent alignment across the entire dataset. If fewer than ten images remained, the macro adapted by merging the available images with the reference stack to avoid misalignment.

To further refine the data for 3D analysis, the images were downscaled by a factor of five to reduce the data load for volumetric rendering. A brain mask was generated using the “Surface Segmentation” tool in Imaris and re-imported into ImageJ. Noise reduction was achieved by overlaying the mask onto the original image stack using the ImageJ Image Calculator, with the “Minimum” function applied to set all noise regions to zero while preserving the smaller structures. A final alignment step using StackReg was then applied to a reversed version of the stack with noise removed, further improving image consistency.

Collectively, these ImageJ custom macros streamline the preprocessing of large histological datasets by automating file renaming, standardizing pixel intensities, aligning datasets across multiple batches, and overlaying images onto a common background.

By integrating machine learning-based segmentation with automated image preprocessing, this pipeline enhances CMH quantification accuracy and scalability, paving the way for efficient screening of cerebrovascular protective interventions.

### Statistical analysis

All data are presented as mean ± standard error of the mean (SEM). The Shapiro–Wilk test was used to assess the normality of data distribution, while the Brown-Forsythe test was applied to evaluate the equality of variances. For comparisons between image analysis methods, one-way analysis of variance (ANOVA) followed by Dunnett’s post hoc test was conducted. For multiple comparison between young, aged, and SS-31-treated aged mice, Kruskal–Wallis non-parametric test was employed. Statistical significance was defined as *p* < 0.05. All statistical analyses were conducted in GraphPad Prism 10.4.1.

## Results

### Quantitative comparison of CMH detection methods

Previously, we demonstrated that color deconvolution combined with manual counting is a viable method for quantifying CMH burden in brain tissue [25, 65, 66]. In this study, we qualitatively and quantitatively compared our newly developed automated quantification pipelines to the gold standard manual counting and color deconvolution-based methods.

**Fig. 2:**
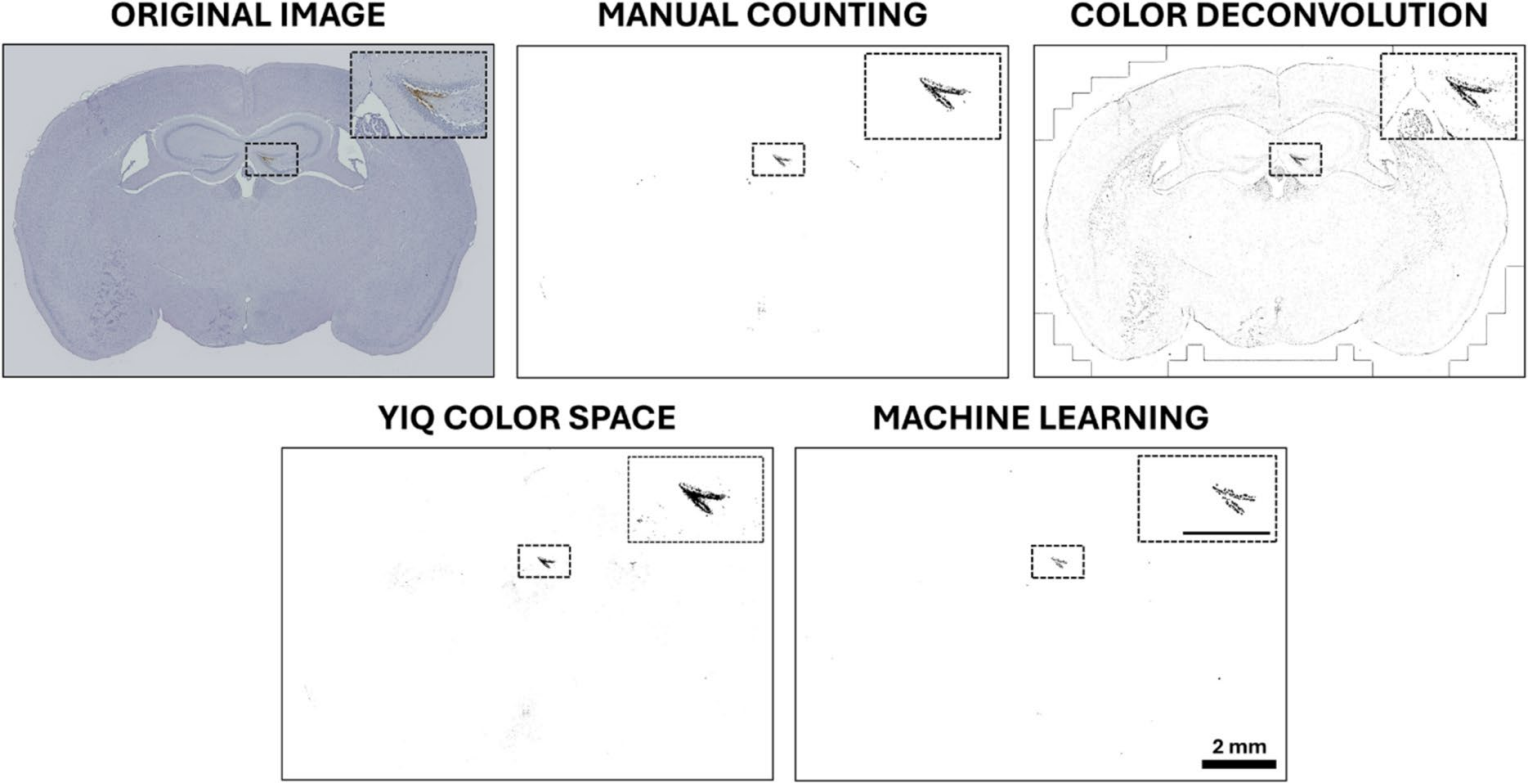
Comparative visualization of CMH detection using different quantification methods. The original coronal brain section image from an aged hypertensive mouse illustrates hematoxylin and diaminobenzidine (DAB) staining, where brain tissue appears blue, and microbleeds are identified by brown DAB precipitates. This representative section highlights a CMH affecting the hippocampus, demonstrating the morphological characteristics of hypertension-induced cerebral microhemorrhages. Segmented images depict the results of different CMH quantification methods, with microbleeds represented as black signals. The YIQ color space transformation demonstrated the closest visual similarity to manual counting, effectively identifying microbleeds with minimal artifacts. In contrast, the color deconvolution method generated numerous artifacts, while the machine learning (ML)–based algorithm underestimated the CMH area, indicating undersampling of smaller microbleeds. Insets highlight the outlined tissue segments marked by black dashed rectangles for enhanced clarity. Inset scalebar: 500 µm. Scalebar: 2 mm

**Fig. 3:**
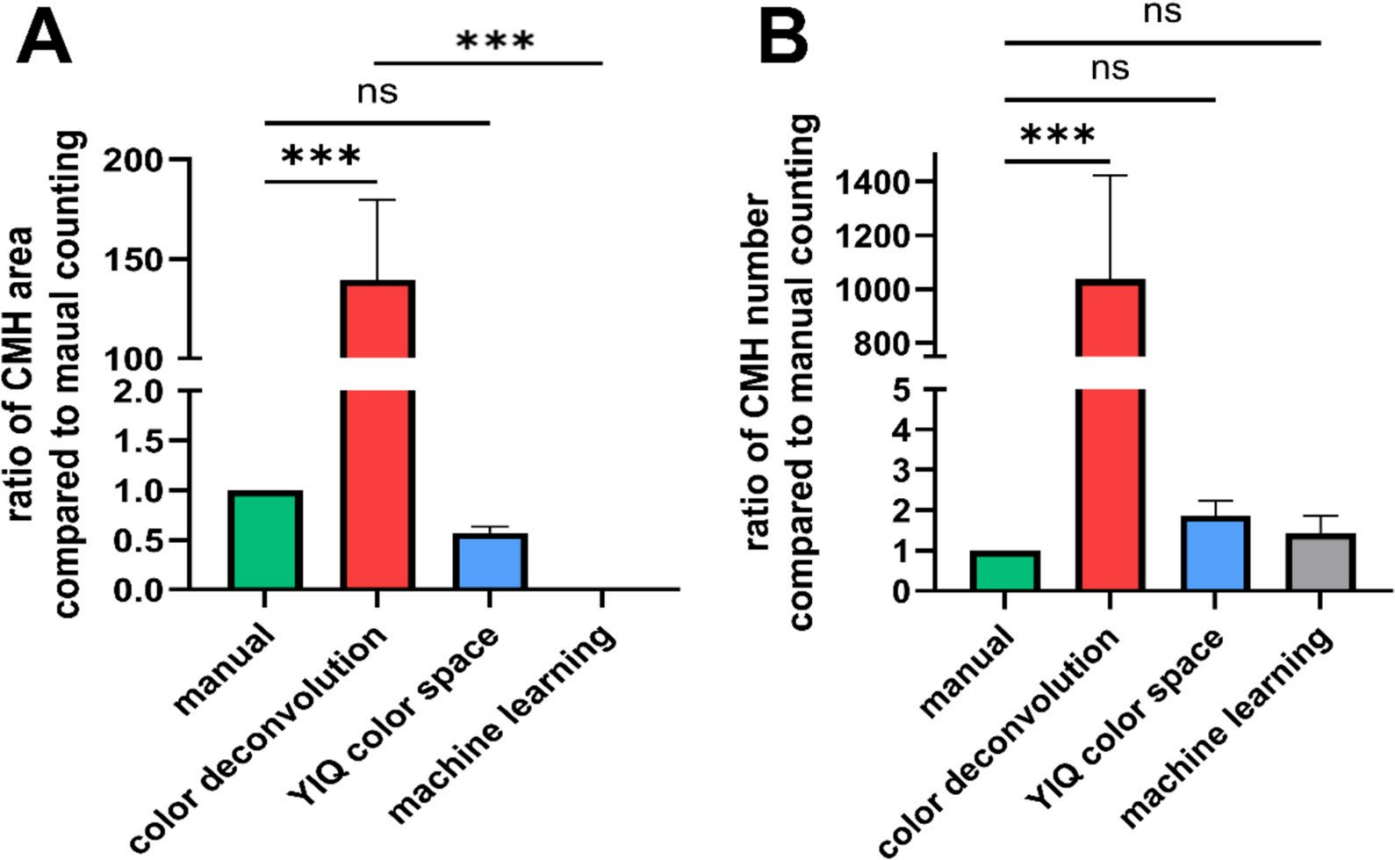
Quantitative comparison of CMH area and count across different analysis methods. Quantitative evaluation of cerebral microhemorrhage (CMH) area (**A**) and CMH count (**B**) is presented as a ratio relative to manual counting to highlight differences between the tested quantification methods. **A** The color deconvolution method significantly overestimated CMH area, likely due to the inclusion of artifacts, while the machine learning (ML)–based model underestimated CMH area, suggesting undersampling of microbleeds. In contrast, the YIQ color space transformation produced results comparable to manual counting, with no significant differences observed. **B** Consistent with CMH area quantification, color deconvolution also overestimated CMH count, while both the color space transformation and ML-based methods reported microbleed numbers similar to manual counting. Data are presented as mean ± SEM. Statistical significance was determined using one-way analysis of variance (ANOVA) followed by Dunnett’s post hoc test. Ns, nonsignificant, ***p < 0.001; *n* = 60 per group

One of our novel approaches replaces color deconvolution with a YIQ color space transformation, which converts images from their native 8-bit indexed or RGB color space into YIQ. This transformation enhances contrast by isolating chrominance and luminance components, improving CMH segmentation and reducing artifacts. Qualitatively, the YIQ transformation provided superior visual clarity compared to other methods (Fig. 2). For quantitative validation, CMH area (Fig. 3A) and CMH count (Fig. 3B) were compared across all methods.

Our analysis revealed that color deconvolution without manual correction led to a 139 ± 40-fold overestimation of CMH area relative to manual counting (Fig. 3A). Conversely, the machine learning (ML)–based classifier underestimated CMH area, producing a 2.93 × 10^−5^ ± 1.3 × 10^-fivefold^ decrease compared to manual counting. The YIQ transformation yielded the most comparable results to manual counting, with a 0.57 ± 0.06 ratio, indicating it provided a balanced estimate of CMH area.

Similarly, CMH number quantification followed the same trend. Color deconvolution significantly overestimated CMH count by 1038 ± 384-fold relative to manual counting (Fig. 3B), while the YIQ transformation (1.8 ± 0.3-fold increase) and ML-based classifier (1.4 ± 0.4-fold increase) produced values much closer to manual quantification (Fig. 3B). Statistical analysis revealed that color deconvolution significantly differed from manual counting in both CMH area (*p* < 0.001) and CMH number (*p* < 0.001), confirming its unsuitability for CMH quantification without manual correction. The ML-based classifier significantly differed in CMH area (*p* < 0.001) but not in CMH number (*p* > 0.99), suggesting its strength lies in CMH count estimation rather than area quantification. The YIQ transformation showed no significant difference from manual counting in either CMH area (*p* > 0.99) or CMH number (*p* > 0.99), confirming it as the most reliable and accurate alternative for CMH quantification.

In addition to accuracy, an essential factor in selecting an optimal quantification method is time efficiency. While an experienced histologist can manually count CMHs with high accuracy, this approach is labor-intensive and time-consuming. To compare the efficiency of different methods, we measured the time required for preprocessing and quantification of CMH-related parameters (Table 1). Compared to manual counting, semi-automated approaches (color deconvolution, color space transformation, and machine learning-based classification) significantly reduced processing time. Among these, YIQ color transformation proved to be the most effective method, reducing time investment by an impressive ~ 98% compared to manual counting while maintaining accuracy comparable to the manual approach.

**Table 1:** Comparative analysis of workload intensity and processing time across CMH quantification methods

Quantification method	Time requirement for different steps in minutes (mean ± SEM)			*P* -value compared to manual counting	Execution
	Preprocessing	Counting	Quantification of a single image		
Manual counting	0.0 ± 0.0	154 ± 10	154 ± 10	–	Manual
Color deconvolution	2.3 ± 0.2	0.1 ± 0.01	2.4 ± 0.2	< 0.001 (***)	Semi-automatic
Color space transformation	0.04 ± 0.01	0.08 ± 0.01	0.12 ± 0.01	< 0.001 (***)	Semi-automatic
Machine learning	0.51 ± 0.03	0.08 ± 0.01	0.59 ± 0.03	< 0.001 (***)	Automatic

Taken together, these findings establish YIQ color space transformation as the most accurate, efficient, and reliable method for CMH quantification. Unlike color deconvolution, which requires extensive manual correction, or machine learning, which struggles with precise area estimation, YIQ transformation offers high accuracy, minimal manual intervention, and substantial time savings, making it the preferred approach for high-throughput CMH analysis.

### Three-dimensional reconstruction for whole-brain CMH burden assessment

Beyond quantitative analysis, three-dimensional (3D) reconstruction enables comprehensive whole-brain CMH burden assessment, providing an anatomical representation of microbleed distribution across brain regions. Custom ImageJ macros and built-in Imaris tools were employed to align segmented brain tissue and CMHs, facilitating high-resolution 3D visualization. This approach enhances the qualitative representation of CMH diversity, capturing variations in size, shape, and spatial distribution across different brain regions.

**Fig. 4:**
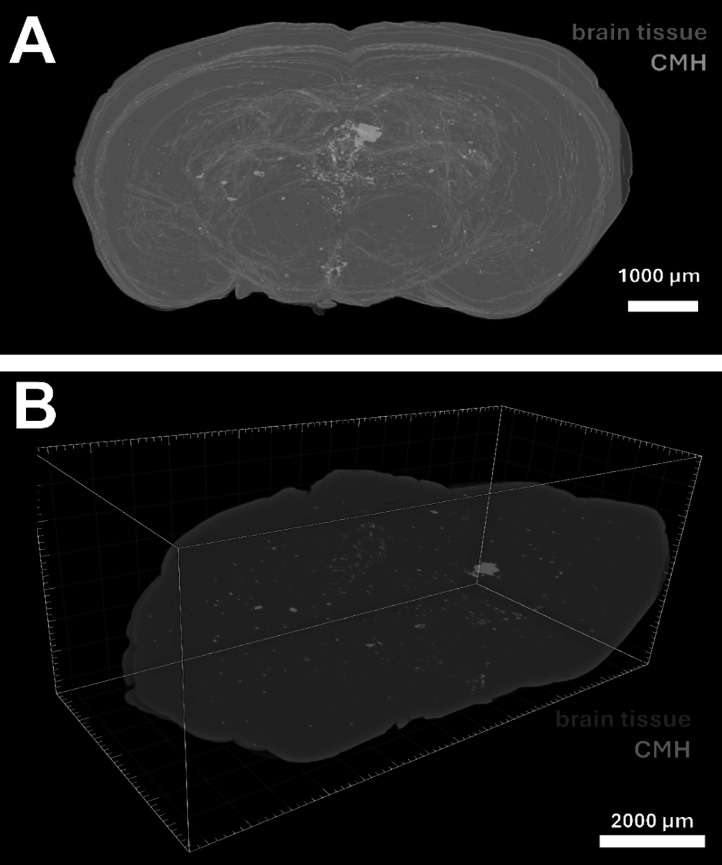
Three-dimensional reconstruction of CMH distribution in the aging brain. **A** Semi-transparent whole-brain reconstruction in a coronal view displaying the anatomical localization of segmented cerebral microhemorrhages (CMHs, pink) relative to brain tissue (blue). Scalebar: 1 mm. **B** Isometric view of the whole-brain reconstruction illustrating the topological distribution of CMHs (pink) along the rostrocaudal axis of the brain tissue (blue). Scalebar: 2 mm

To ensure fully automated CMH identification and quantification, a ML-based segmentation approach was implemented. Unlike color deconvolution and color space transformation, which require semi-automated processing, the ML model processed all images autonomously after training. To standardize the image dataset, image dimensions were normalized and consistently centered. Alignment was performed iteratively to minimize displacement between consecutive image sets (Supplementary image file 1). The images were subsequently downscaled to accommodate three-dimensional rendering constraints. Noise was effectively removed while preserving finer brain structures using a combination of three-dimensional surface masking and pixel-based filtering (Supplementary image file 2). This automated and scalable workflow enabled the creation of detailed whole-brain CMH reconstructions, enhancing the spatial understanding of microbleed distribution (Fig. 4).

While segmentation is highly efficient, taking only ~ 0.5 min per image (Table 1), whole-brain reconstruction, alignment, and rendering remain computationally intensive. However, this step is fully automated and does not require manual supervision. The speed of reconstruction depends on hardware capabilities. Using a high-performance workstation equipped with an AMD Ryzen Threadripper PRO 3975WX 3.50 GHz processor, 64GB DDR4 3200MHz RAM, and an NVIDIA RTX A4000 16GB GDDR6 GPU, the system can fully reconstruct one brain per day.

### Effect of SS-31 treatment on hypertension-induced CMH burden in aged mice

Having established a fast and reliable quantification pipeline for CMH burden assessment, we applied this method to evaluate the effect of SS-31 treatment on hypertension-induced CMH formation in aged mice. Consistent with qualitative observations, a marked increase in microbleed formation was observed in aged hypertensive mice compared to young controls (Fig. 2). However, no visible reduction in CMH burden was detected in SS-31-treated aged hypertensive mice based on visual assessment.

**Fig. 5:**
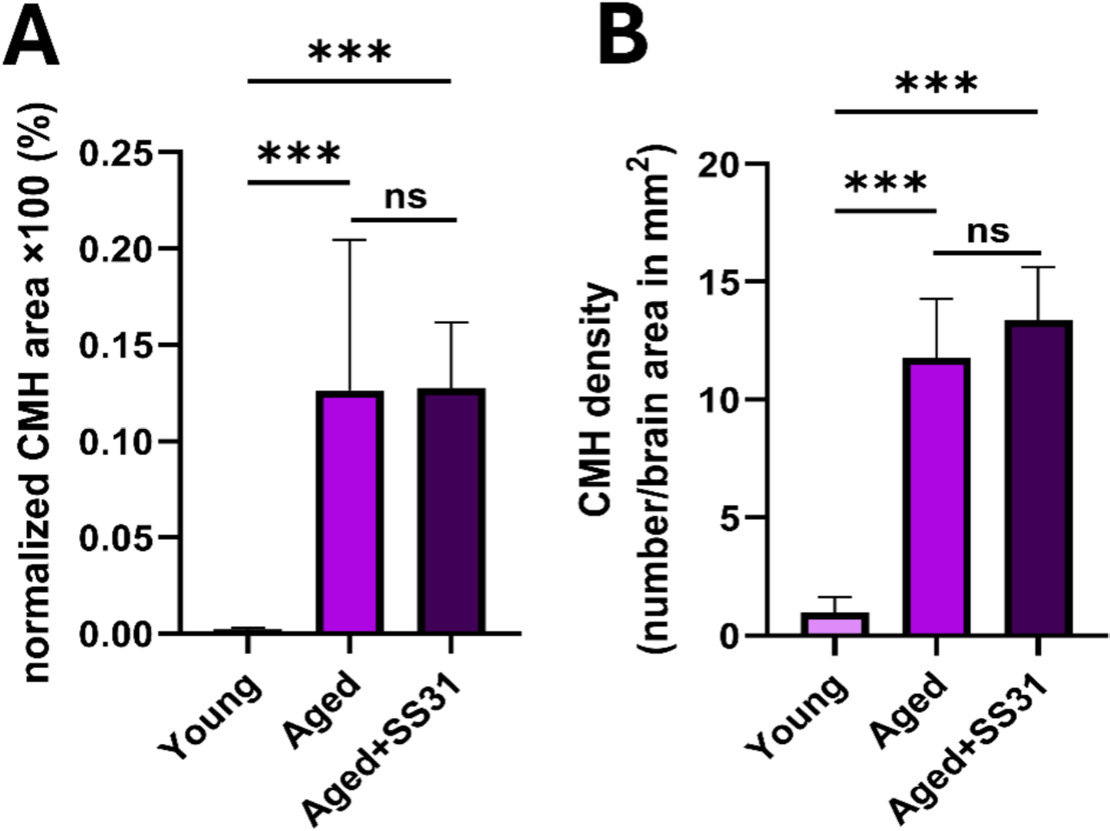
SS-31 treatment fails to reduce hypertension-induced CMH burden in aged mice. **A** Normalized CMH area was significantly increased in aged hypertensive mice compared to young controls. However, SS-31 treatment did not mitigate CMH formation in aged hypertensive mice. **B** CMH density, normalized to a 1-mm^2^ brain area, was significantly higher in the aged hypertensive group compared to young controls, with no reduction observed in SS-31-treated mice. These findings indicate that SS-31 treatment fails to attenuate hypertension-induced CMH formation in aged mice, suggesting that targeting mitochondrial oxidative stress alone may not be sufficient to prevent microbleed development. Data are presented as mean ± SEM. Statistical significance was assessed using the Kruskal–Wallis nonparametric test, followed by Dunnett’s post hoc pairwise comparison. **ns: nonsignificant, *p < 0.001; *n* = 3 brains per group

To validate these observations, color space transformation–based quantification was performed (Fig. 5). Normalized CMH area was significantly elevated (*p* < 0.001) in aged hypertensive mice (0.13% ± 0.08%) compared to young controls (2.16 × 10^−3^% ± 1.1 × 10^−3^%). Similarly, CMH density, normalized to a 1-mm^2^ brain area, was substantially higher in aged hypertensive mice (11.79 ± 2.50) compared to young controls (0.98 ± 0.65, *p* < 0.001). SS-31 treatment did not significantly alter CMH burden in aged hypertensive mice, with CMH area (0.13% ± 0.03%) and microbleed density (13.39 ± 2.42) remaining comparable to untreated aged hypertensive controls. These findings indicate that SS-31 treatment did not mitigate CMH formation in aged hypertensive mice. Despite its known mitochondrial protective effects, SS-31 failed to reduce hypertension-induced cerebromicrovascular damage, suggesting that additional or alternative therapeutic strategies may be necessary to counteract microvascular fragility in aging.

## Discussion

Our study provides a novel, high-throughput approach for detecting and quantifying CMHs, allowing for a robust evaluation of potential cerebromicrovascular protective interventions. Despite the known mitochondrial protective effects of SS-31 [37, 58–62], we found no significant reduction in CMH burden in aged hypertensive mice treated with SS-31. These findings suggest that targeting mitochondrial oxidative stress alone may be insufficient to prevent hypertension-induced CMH formation, highlighting the multifactorial nature of CMH pathogenesis.

Although SS-31 is a potent mitochondrial-targeted antioxidant, our findings suggest that mitochondrial oxidative stress alone is not the sole driver of hypertension-induced CMH formation in the aging cerebral microcirculation [6, 29, 67, 68]. Additional factors, such as increased penetration of high pressure to the thin-walled microvessels due to age-related impairment of myogenic autoregulation [69, 70], impaired IGF-1 signaling [32, 71], chronic inflammation, increased ROS production by non-mitochondrial sources, endothelial senescence, and related extracellular matrix degradation, impaired functional and structural adaptation to hypertension [69, 70, 72, 73] may contribute to microvascular fragility [6, 29, 74–76]. While SS-31 effectively reduces mitochondrial ROS production and stabilizes mitochondrial membrane integrity, it does not directly modulate vascular remodeling, MMP activation, or BBB permeability, which are all implicated in CMH pathogenesis [37, 58, 77]. These results highlight the complexity of CMH development and suggest that mitochondrial-targeted therapies alone may be insufficient for complete protection.

A key limitation of this study is that only male mice were used. Sex differences in vascular aging have been reported, with evidence suggesting that estrogen may exert protective effects in females [78]. However, the primary objective of this study was to evaluate SS-31 in a well-characterized preclinical model while minimizing potential sex-dependent variability. Future studies should explore sex-specific differences in SS-31 efficacy and CMH pathogenesis by incorporating both male and female animals. The duration of SS-31 treatment was selected based on previous studies demonstrating rapid mitochondrial functional improvements and cerebrovascular protection with this peptide [58]. However, in the context of advanced aging, a longer treatment regimen may be required to observe significant cerebrovascular protection against hypertension-induced rupture. The cumulative damage in aged mice may not be sufficiently reversed within this timeframe. Future studies should investigate extended SS-31 administration and assess whether prolonged treatment confers greater cerebrovascular benefits.

Given the multifactorial nature of CMH pathogenesis, a combinatorial treatment strategy may be more effective. Future studies should explore whether SS-31 enhances cerebrovascular resilience when used in conjunction with senolytic agents, MMP inhibitors, or anti-inflammatory compounds. For example, senolytics may mitigate endothelial senescence, MMP inhibitors could prevent excessive extracellular matrix degradation, and anti-inflammatory interventions may help preserve microvascular integrity. Investigating such combination therapies may provide a more comprehensive approach to preventing CMH formation and reducing cerebrovascular injury in aging.

Our study also underscores the importance of using robust, scalable, and unbiased image analysis techniques for CMH quantification. Our results demonstrate that manual counting, while considered the gold standard, is highly time-consuming. The color deconvolution method significantly overestimated the CMH burden, making it less reliable without manual correction. In contrast, YIQ color space transformation provided results that closely matched manual counting while reducing processing time by over 98%, making it the most suitable method for high-throughput analysis. The ML-based approach using a random forest classifier showed promise for CMH detection, particularly in counting smaller microbleeds. However, it exhibited inferior accuracy in quantifying CMH area, likely due to its reliance on predefined training data that may not fully capture the heterogeneity of CMH morphology. Future improvements, such as implementing deep learning models (e.g., convolutional neural networks), could enhance segmentation precision and adaptability across different staining techniques and imaging conditions. Taken together, the image analysis pipeline developed here significantly improved detection accuracy and reduced processing time, making it an invaluable tool for future preclinical screening of potential therapeutic interventions. Beyond CMH detection, our high-throughput machine learning-driven imaging pipeline can be adapted to analyze additional histological markers of microvascular pathology. The segmentation framework is highly flexible and can be trained to identify vascular amyloid deposits, fibrinogen leakage, ischemic lesions, or other biomarkers associated with cerebrovascular aging and neurovascular dysfunction. With further optimization, this pipeline could be applied to a range of preclinical studies assessing cerebrovascular pathology across different disease models, further expanding its utility in aging and neurovascular research.

In conclusion, our findings emphasize the complexity of CMH pathogenesis and highlight the need for multi-targeted therapeutic approaches. While SS-31 did not reduce CMH burden, our high-throughput imaging pipeline represents a critical advancement in CMH research and will facilitate future investigations into cerebromicrovascular protective strategies.

## Supplementary Information

Below is the link to the electronic supplementary material.

Supplementary file1 Validation of the boundary alignment through coronal plane Boundary alignment was validated through sequential coronal plane imaging, performed iteratively to minimize displacement between consecutive image sets. The sequential cross-sectional imaging along the coronal view revealed no visible tissue displacement throughout the rostrocaudal axis, starting from the anterior anatomical structures (olfactory bulb) and extending to the posterior regions (cerebellum and brainstem). This consistent alignment confirms proper tissue registration and image stitching across the dataset. (MP4 4531 KB)

Supplementary file2 3D representation of the cerebral microbleeds in the brain volume A three-dimensional isometric view illustrates the alignment of whole-brain tissue (blue) with segmented cerebral microbleeds (pink). This visualization demonstrates the feasibility of the newly developed method to segment microbleeds with anatomically precise localization, enabling the assessment of CMH topology under various pathological conditions. Additionally, it provides a robust framework for evaluating the efficacy of pharmacological interventions aimed at mitigating microbleed formation. (MP4 22207 KB)

## Data Availability

The data supporting the findings of this study can be found either in the main text or can be obtained from the authors upon a reasonable request.
